# Body mass index is positively associated with flatfoot and inversely associated with hindfoot valgus in school-aged children: a cross-sectional analysis of 3,292 screening records

**DOI:** 10.3389/fped.2026.1930830

**Published:** 2026-09-09

**Authors:** Shanyuan Li, Dongmei Li, Yongxin Huo, Ying Xu, Yuhang Wang, Hongyan Zhou, Xinyang Dong, Ying Song, Weixin Yang, Bin Wang

**Affiliations:** 1 Department of Rehabilitation, Tangshan Second Hospital, Tangshan, Hebei, China; 2 Institute of Traumatology, Tangshan Second Hospital, Tangshan, Hebei, China; 3 Tianjin Delijie Medical Devices Co., Ltd., Tianjin, China

**Keywords:** body mass index, flatfoot, hallux valgus, hindfoot valgus, pediatric orthopedics, school screening

## Abstract

**Background:**

A higher body mass index (BMI) has been associated with pediatric flatfoot, but it is unclear whether BMI relates similarly to arch morphology, hallux alignment, and hindfoot alignment when these measures are collected within the same school-screening workflow. Herein, we examined phenotype-specific associations between BMI and three scanner-derived foot measures.

**Methods:**

This secondary cross-sectional analysis linked project-based school foot-screening records to on-site anthropometric measurements. After linkage and quality-control exclusions, 3,292 records from six primary schools were analyzed. Record-level outcomes were moderate-to-severe flatfoot (arch index ≥ 0.28), mild hallux valgus (15° to <30°), and moderate-to-severe hindfoot valgus (≥4°), with positivity defined by either foot. BMI was analyzed in sample-specific quartiles and per 1 internal SD. Multivariable binary logistic regression adjusted for age, sex, and school; threshold and duplicate-identifier sensitivity analyses were performed.

**Results:**

The 3,292 records represented 3,266 unique school-student identifiers; median age was 10 years (IQR: 9–12), and There were 1,616 female records (49.1%). Screening prevalence was 64.3% for flatfoot, 11.5% for mild hallux valgus, and 52.8% for hindfoot valgus. Each 1-SD higher BMI (4.73 kg/m^2^) was associated with higher odds of flatfoot [odds ratio (OR): 1.37, 95% CI: 1.26–1.49; *P* < 0.001] and lower odds of hindfoot valgus (OR: 0.82, 95% CI: 0.76–0.88; *P* < 0.001), while mild hallux valgus showed no statistically significant association (OR: 0.93, 95% CI: 0.82–1.05; *P* = 0.244). More stringent threshold analyses and analyses retaining one record per identifier produced materially similar BMI associations.

**Conclusions:**

BMI was positively associated with flatfoot and inversely associated with hindfoot valgus, while no significant association was observed for mild hallux valgus. These cross-sectional associations describe operational scanner-derived screening phenotypes and should not be interpreted as causal effects or clinical diagnoses. Prospective studies with documented anthropometric quality control and validated clinical or radiographic measures are needed.

## Introduction

Both body mass and foot morphology change substantially during childhood. Body mass index (BMI) references are useful for growth surveillance, while the developing foot is influenced by skeletal maturation, loading, soft-tissue thickness, ligamentous properties, and postural control (1, 2). Pediatric flatfoot is common and usually flexible, but prevalence estimates vary because clinical examination, radiography, footprints, and plantar-pressure methods quantify different constructs (3, 4). The footprint-derived arch index offers a practical quantitative measure for field screening (5).

A higher BMI has repeatedly been associated with pediatric flatfoot and lower arch measures. Systematic reviews, footprint studies, and plantar-pressure investigations suggest contributions from mechanical loading and increased plantar soft-tissue contact, although results depend on age, sampling, and the measurement definition used (6–9). Studies of pediatric arch morphology have also identified associations with age, sex, joint laxity, footprint classification, and hindfoot alignment (10–14), and recent school-based studies continue to show that prevalence varies across populations and criteria (15, 16).

Evidence is less developed for BMI in relation to hallux valgus and hindfoot valgus. Pediatric hallux valgus has developmental and anatomic determinants that differ from those of flatfoot (17–20), while hindfoot angle measures calcaneal alignment rather than plantar arch contact (21). Operational cutoffs also vary across instruments and studies, and no single scanner threshold is a universal pediatric diagnosis. Consequently, combining arch, hallux, and hindfoot findings into one “foot abnormality” outcome may conceal phenotype-specific patterns.

We therefore linked anthropometric rosters with project-based static foot-scanner records from participating primary schools. The objective was to estimate the prevalence of moderate-to-severe flatfoot, mild hallux valgus, and moderate-to-severe hindfoot valgus and to examine their adjusted associations with BMI within a common screening workflow. We hypothesized that a higher BMI would be positively associated with flatfoot and that associations with hallux and hindfoot alignment would not necessarily follow the same direction. Because the data were cross-sectional and the thresholds were operational, all outcomes were interpreted as screening phenotypes rather than clinical diagnoses.

## Materials and methods

### Study design, setting, and data linkage

This secondary cross-sectional study used deidentified records generated during school-based foot-health screening conducted within the broader research project “Motor Function Assessment and Intelligent Rehabilitation of Foot Diseases in Primary and Secondary School Students in Tangshan.” In the analytic file, direct personal identifiers were removed and schools were represented by anonymous codes; identifiers required for linkage and quality control were retained only in restricted files. The seven primary schools participating in the parent project defined the source dataset of 3,634 foot-scanner records in Tangshan, Hebei Province, China. Within these schools, all available screening records meeting the original participant-level eligibility criteria were considered, with no individual-level subsampling.

### Eligibility, linkage, and sample size

The original screening protocol excluded children with generalized joint hypermobility, ligamentous laxity, previous foot or lower-limb injury, other congenital or acquired foot or lower-limb deformities, or markedly atypical physical activity levels. These factors were addressed through participant selection rather than entered as model covariates. Among eligible children, foot records were linked to school rosters by school and normalized student identifier. A linkage was accepted when recorded sex agreed and ages differed by no more than 1 year. Anthropometric values were accepted when height was 100–190 cm, weight was 15–120 kg, and calculated BMI was 8–45 kg/m^2^. Records were excluded when no roster match was available, demographic agreement failed, or anthropometry/BMI was outside these quality-control ranges. Of 3,634 source records, 245 were unmatched, 90 had demographic conflicts, and 7 had invalid anthropometry or BMI after demographic agreement, leaving 3,292 records from six schools. One source school had no linkable anthropometric roster and contributed no records to the BMI analysis. The final records covered ages 6–14 years and both sexes.

Sample-size adequacy for prevalence estimation was assessed using the single-proportion formula *n* = *Z*^2^*p*(1 − *p*)/*d*^2^. With a two-sided 95% confidence level (*Z* = 1.96), a conservative expected prevalence rate of 50% (*p* = 0.50), and absolute precision of 2 percentage points (*d* = 0.02), the required analyzable sample was 2,401 records. Allowing for up to 20% unlinked or unusable records yielded a source-record requirement of 3,002 [2,401/(1–0.20), rounded up]. The 3,634 source records and 3,292 analyzable records exceeded these values. All records meeting the participant-level eligibility, linkage, and quality-control criteria were included; no individual-level subsampling was performed.

### Ethics approval

The study protocol was reviewed and approved by the Medical Ethics Committee of Tangshan Second Hospital (approval no. 2024-71; approval date: 18 September 2024). The present analysis used deidentified school screening records, and only anonymous school codes were used in the manuscript and figures. Written informed consent for participation was not required from participants or their parents/legal guardians in accordance with national legislation and institutional requirements, as approved by the ethics committee.

### Anthropometry

Height (cm) and weight (kg) were measured on site during the school screening visits using internationally standardized anthropometric procedures (22) and a Shanghe SH-200 height-and-weight measuring instrument (Zhengzhou Shanghe Electronic Technology Co., Ltd., Zhengzhou, China). The measured values were recorded in the school screening rosters and linked to the foot-screening records by school and student identifier.

### Screening phenotypes

According to the source screening protocol, bilateral static weight-bearing foot scans were obtained using a foot analysis instrument (model DLJYXSMYBD01; Tianjin Delijie Medical Devices Co., Ltd., Tianjin, China). After excluding the toes, each plantar footprint was divided into equal thirds along its longitudinal axis. Arch index was calculated as AI = *B*/(*A* + *B* + *C*), where *A*, *B*, and *C* denote forefoot, midfoot, and rearfoot contact areas, respectively (5). Bilateral scanner–derived hallux deviation and hindfoot angles were also recorded. The project report defined the hindfoot angle as the angle between the longitudinal bisectors of the lower leg and calcaneus; further details of landmark identification and software processing were unavailable.

To reproduce the primary spreadsheet classifications, a record was positive when either foot met the following operational thresholds: arch index ≥0.28 for moderate-to-severe flatfoot, hallux angle 15° to <30° for mild hallux valgus, or hindfoot angle ≥4° for moderate-to-severe hindfoot valgus. Bilateral positivity was counted once at the record level and the two feet were not modeled separately. These thresholds were treated as screening rules rather than universally accepted pediatric diagnostic criteria (3, 4, 14, 18). The source narrative instead began hallux valgus at 16° and described ≤4° as normal hindfoot alignment; both alternative boundaries were examined in sensitivity analyses.

The manufacturer reported having conducted postproduction inter-rater and intra-rater reliability assessments. However, the assessment methods and numerical results have not been publicly released and could not be independently appraised. No publicly available radiographic criterion-validity data for this device model were identified. Reliability reported for the foundational footprint arch-index method under standardized conditions does not constitute device-specific validation of the scanner or its angle algorithms.

### BMI exposure and statistical analysis

BMI was calculated as weight in kilograms divided by squared height in meters. The primary exposure was continuous BMI standardized using the analytic-sample mean and SD; 1 SD corresponded to 4.73 kg/m^2^. Sample-specific BMI quartiles were used only for descriptive analyses. Because dates of birth were unavailable and age was recorded only in completed years, exact age in months could not be derived reliably. Sex-specific WHO BMI-for-age z scores and weight-status categories therefore could not be calculated reliably. The internal SD and quartiles indicate relative BMI within this sample, are not clinical weight-status categories, and have sample-dependent cut points that may limit comparisons with other populations. Outcomes were binary record-level indicators. Multivariable binary logistic regression included standardized BMI, age as a continuous variable, sex (male = 1; female reference), and five school fixed-effect indicators (School A reference). School coefficients were included in each model but omitted from Table 1 for concision. Odds ratios (ORs), 95% confidence intervals, and two-sided *P* values were reported, with *P* < 0.05 considered statistically significant. Overall and stratum-specific prevalence was summarized with Wilson 95% confidence intervals. Model fit was assessed using McFadden pseudo-*R*^2^, the Akaike information criterion, and the Hosmer–Lemeshow test based on deciles of fitted probability. Model-standardized BMI-response curves averaged predicted probabilities over the observed age, sex, and school distribution. Sensitivity analyses applied the source narrative boundaries of 16° to <30° for hallux valgus and >4° for hindfoot valgus and retained only the first record for each school-student identifier. Analyses were conducted in Python (version 3.11.5).

**Table 1:** Multivariable binary logistic regression models.

Screening phenotype	Predictor	OR (95% CI)	*P*-value
Moderate-to-severe flatfoot	BMI, per 1 SD	1.37 (1.26–1.49)	<0.001
Moderate-to-severe flatfoot	Age, per year	0.74 (0.69–0.78)	<0.001
Moderate-to-severe flatfoot	Male (reference: female)	1.77 (1.52–2.05)	<0.001
Mild hallux valgus	BMI, per 1 SD	0.93 (0.82–1.05)	0.244
Mild hallux valgus	Age, per year	1.20 (1.09–1.31)	<0.001
Mild hallux valgus	Male (reference: female)	0.33 (0.26–0.41)	<0.001
Moderate-to-severe hindfoot valgus	BMI, per 1 SD	0.82 (0.76–0.88)	<0.001
Moderate-to-severe hindfoot valgus	Age, per year	0.91 (0.86–0.96)	0.001
Moderate-to-severe hindfoot valgus	Male (reference: female)	1.38 (1.20–1.59)	<0.001

ORs are from binary logistic regression models including all displayed predictors and five school fixed-effect indicators; School A was the reference and school coefficients are omitted for concision. Female was the sex reference. BMI was standardized within the analytic sample; 1 SD = 4.73 kg/m^2^. Outcomes were positive when either foot met the primary operational threshold. Model fit for flatfoot, mild hallux valgus, and hindfoot valgus, respectively: McFadden pseudo-*R*^2^=0.049, 0.055, and 0.017; AIC=4,100.1, 2,239.7, and 4,493.4; Hosmer–Lemeshow *P* = 0.276, 0.339, and 0.732. All *P*-values are two-sided.

## Results

### Data linkage and analytic sample

**Figure 1:**
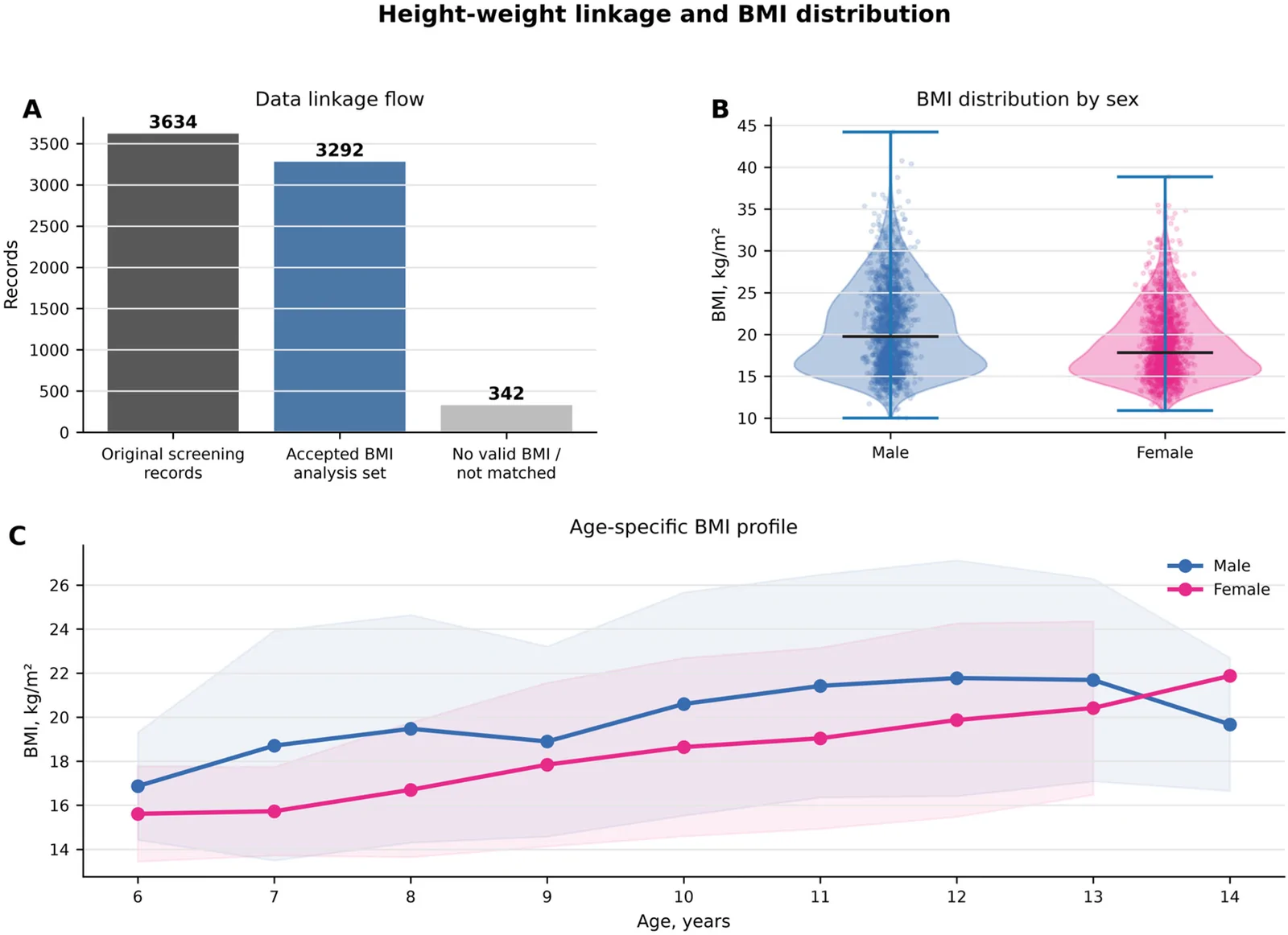
Height-weight linkage and BMI distribution. **(A)** Linkage flow from 3,634 source foot-screening records to 3,292 BMI-linked analytic records; 342 exclusions comprised 245 unmatched records, 90 demographic conflicts, and seven invalid anthropometry/BMI records. **(B)** BMI distribution by sex. **(C)** Age-specific BMI profile. The analytic records represented 3,266 unique school-student identifiers.

Of the 3,634 source foot-screening records, 245 could not be linked to an anthropometric roster, 90 had discordant sex or age between sources, and seven had invalid anthropometry or calculated BMI after demographic agreement, leaving 3,292 records from six schools (Table 2; Figure 1). School-specific sample sizes ranged from 13 to 1,251 records. The analytic records represented 3,266 unique school-student identifiers; 52 records belonged to 26 repeated identifier pairs. Ages ranged from 6 to 14 years, with a median of 10 years (IQR: 9–12). There were 1,616 female records (49.1%) and 1,676 male records (50.9%). Mean BMI was 19.72 kg/m^2^ (SD: 4.73), and median BMI was 18.72 kg/m^2^ (IQR: 16.02–22.74). The BMI quartiles contained 826, 820, 823, and 823 records, respectively.

**Table 2:** Record selection and analytic sample characteristics.

Characteristic	Value
Source foot-screening records	3,634
Unmatched with anthropometric roster	245
Demographic conflicts between sources	90
Invalid anthropometry/BMI after demographic agreement	7
Records included in BMI-linked analysis	3,292
Unique school-student identifiers	3,266
Schools contributing to analysis	6
School A, *n* (%)	1,251 (38.0)
School B, *n* (%)	917 (27.9)
School C, *n* (%)	841 (25.5)
School D, *n* (%)	147 (4.5)
School F, *n* (%)	123 (3.7)
School G, *n* (%)	13 (0.4)
Age range, years	6–14
Age, median (IQR), years	10 (9–12)
Female, *n* (%)	1,616 (49.1)
Male, *n* (%)	1,676 (50.9)
BMI, mean (SD), kg/m^2^	19.72 (4.73)
BMI, median (IQR), kg/m^2^	18.72 (16.02–22.74)
BMI Q1 (≤16.02 kg/m^2^), *n* (%)	826 (25.1)
BMI Q2 (>16.02–18.72 kg/m^2^), *n* (%)	820 (24.9)
BMI Q3 (>18.72–22.74 kg/m^2^), *n* (%)	823 (25.0)
BMI Q4 (>22.74 kg/m^2^), *n* (%)	823 (25.0)

Values are screening records, unless otherwise indicated. Linkage required agreement in sex and an age difference of no more than 1 year. Anthropometry was accepted at height 100–190 cm, weight 15–120 kg, and BMI 8–45 kg/m^2^. School E had 136 source records but no linkable anthropometric roster and contributed no analytic records. BMI quartiles were defined within the analytic sample; unequal counts reflect tied values at the first boundary.

### Prevalence across BMI strata

**Figure 2:**
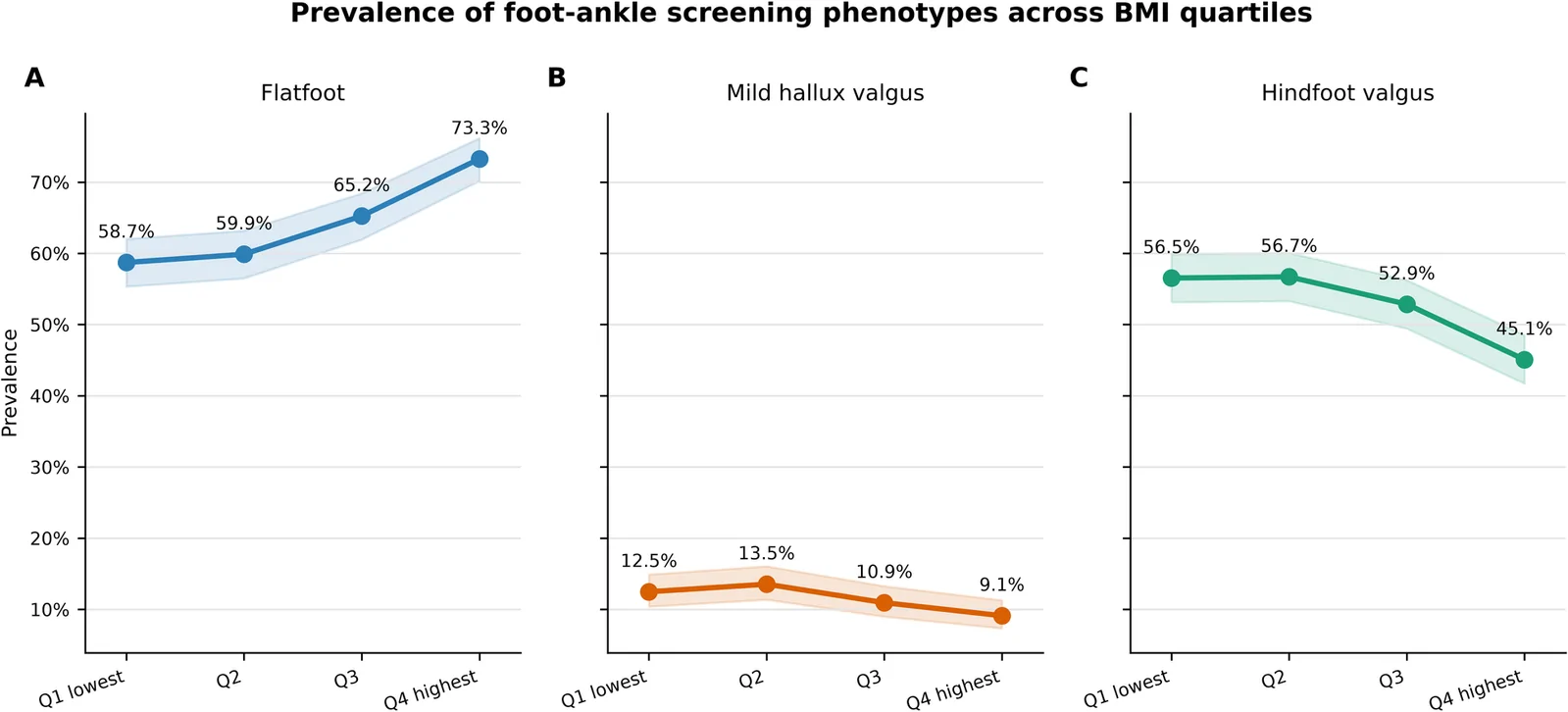
Prevalence of the three foot-ankle screening phenotypes across sample-specific BMI quartiles. **(A)** Moderate-to-severe flatfoot; **(B)** mild hallux valgus; **(C)** moderate-to-severe hindfoot valgus. The points show record-level prevalence and the shaded bands show Wilson 95% confidence intervals. Quartile counts were 826, 820, 823, and 823; unequal counts reflect tied BMI values at the first boundary.

Within the BMI-linked analytic sample, moderate-to-severe flatfoot was present in 2,116/3,292 records (64.3%), mild hallux valgus in 379/3,292 (11.5%), and moderate-to-severe hindfoot valgus in 1,738/3,292 (52.8%) (Table 3). Flatfoot was more prevalent in male than female records (71.5% vs. 56.7%), whereas mild hallux valgus was more prevalent in female records (17.0% vs. 6.2%). Across BMI quartiles, the flatfoot prevalence rate increased from 58.7% to 73.3%, the hindfoot valgus prevalence rate decreased from 56.5% to 45.1%, and the mild hallux valgus rate varied from 12.5% to 9.1% without a monotonic gradient (Table 3; Figure 2). Age-group-specific prevalence rates are also given in Table 3.

**Table 3:** Screening phenotype prevalence by sex, age group, and BMI quartile.

Stratum (*N*)	Moderate-to-severe flatfoot, *n* (%)	Mild hallux valgus, *n* (%)	Moderate-to-severe hindfoot valgus, *n* (%)
Overall (3,292)	2,116 (64.3)	379 (11.5)	1,738 (52.8)
Female (1,616)	917 (56.7)	275 (17.0)	806 (49.9)
Male (1,676)	1,199 (71.5)	104 (6.2)	932 (55.6)
Age 6–8 years (193)	131 (67.9)	13 (6.7)	102 (52.8)
Age 9–11 years (2,166)	1,460 (67.4)	232 (10.7)	1,194 (55.1)
Age 12–14 years (933)	525 (56.3)	134 (14.4)	442 (47.4)
BMI Q1 (826)	485 (58.7)	103 (12.5)	467 (56.5)
BMI Q2 (820)	491 (59.9)	111 (13.5)	465 (56.7)
BMI Q3 (823)	537 (65.2)	90 (10.9)	435 (52.9)
BMI Q4 (823)	603 (73.3)	75 (9.1)	371 (45.1)

Phenotypes were record-level indicators positive when either foot met the primary operational threshold. Percentages use the *N* shown for each stratum. BMI quartiles were sample-specific: Q1 ≤ 16.02, Q2 > 16.02–18.72, Q3 > 18.72–22.74, and Q4 > 22.74 kg/m^2^. Wilson 95% confidence intervals were calculated for all estimates.

### Adjusted BMI associations

**Figure 3:**
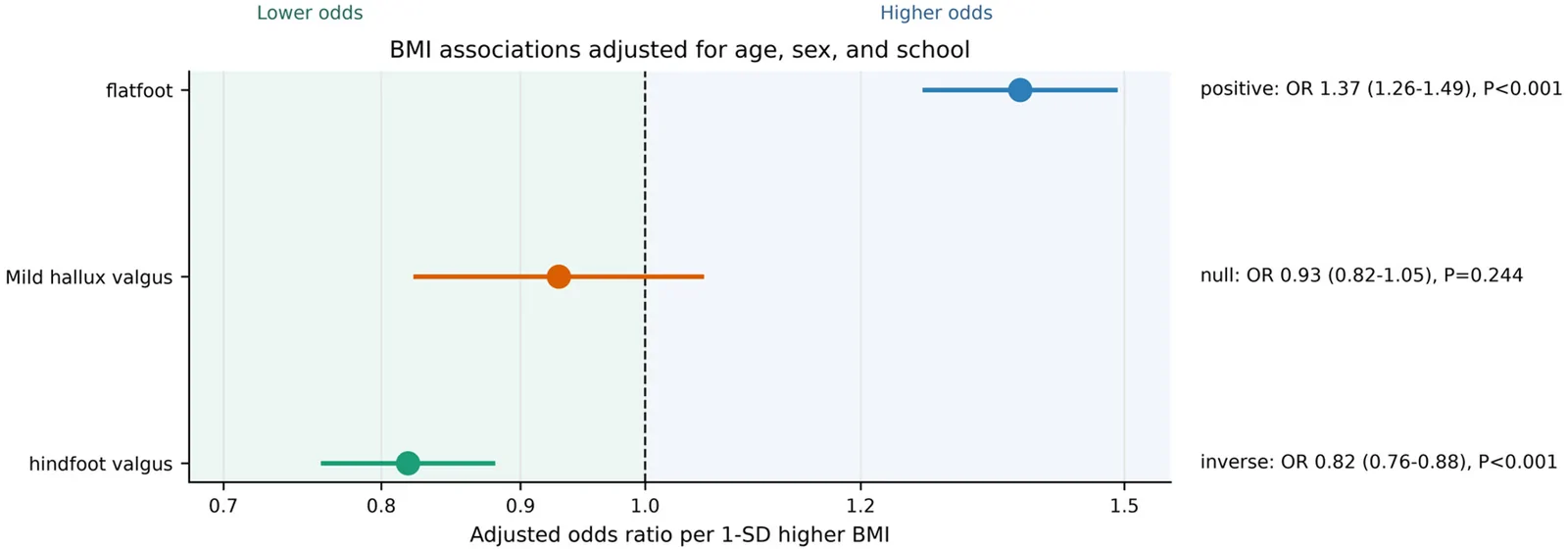
Adjusted odds ratios and 95% confidence intervals for each phenotype per 1-SD higher BMI (4.73 kg/m^2^). Estimates are from multivariable binary logistic regression adjusted for age, sex, and school fixed effects; female and School A were the reference categories.

**Figure 4:**
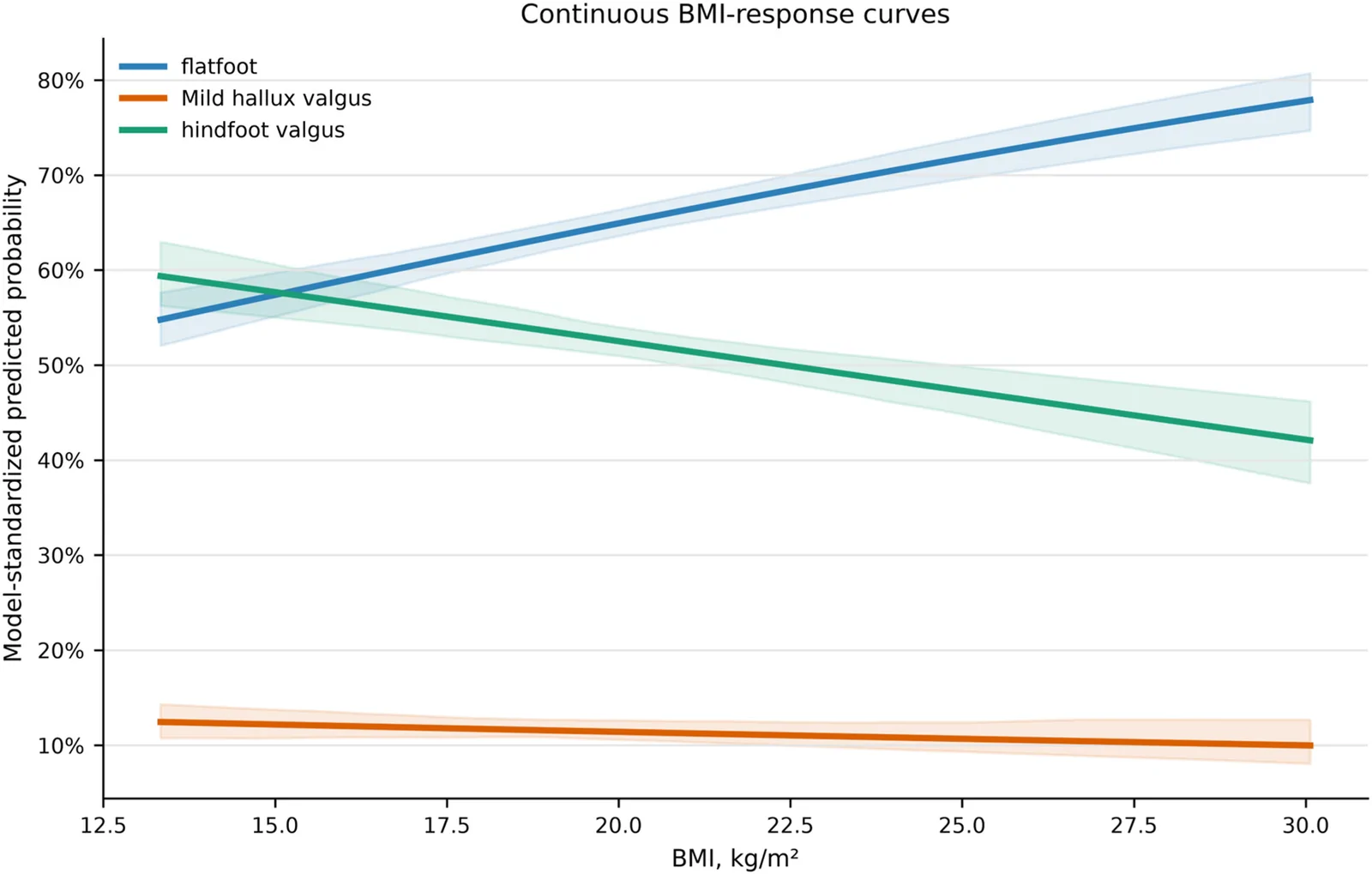
Model-standardized continuous BMI-response curves for the three screening phenotypes. The curves average predicted probabilities over the observed age, sex, and school distribution; the shaded bands show 95% confidence intervals. The curves describe cross-sectional associations and are not WHO BMI-for-age categories.

**Figure 5:**
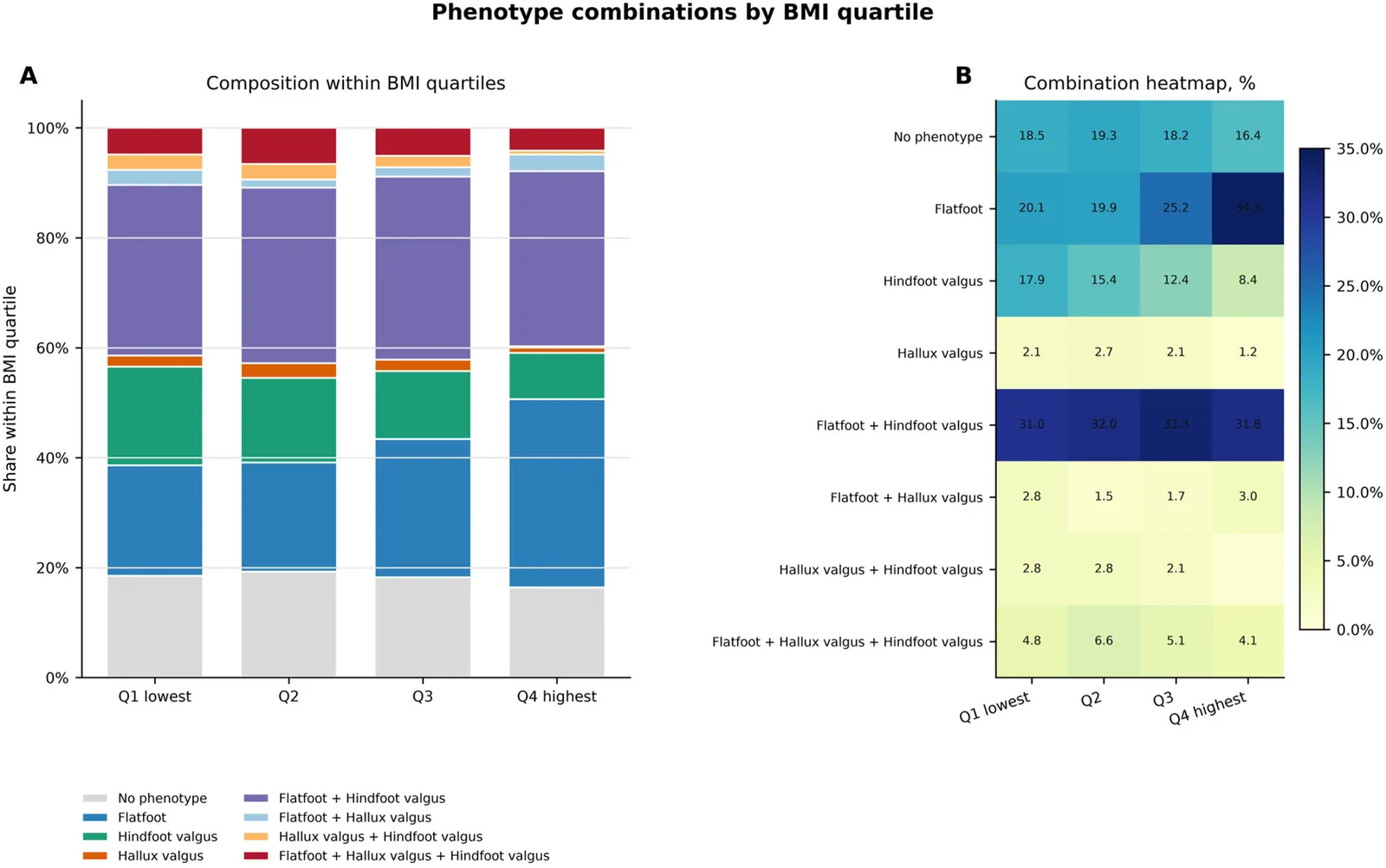
Mutually exclusive phenotype combinations across sample-specific BMI quartiles. **(A)** Stacked percentage distribution within each quartile. **(B)** A heatmap of the same combination percentages. Phenotypes were record-level indicators positive when either foot met the operational screening threshold.

After adjustment for age, sex, and school, each 1-SD higher BMI was associated with higher odds of moderate-to-severe flatfoot (OR: 1.37, 95% CI: 1.26–1.49; *P* < 0.001) and lower odds of moderate-to-severe hindfoot valgus (OR: 0.82, 95% CI: 0.76–0.88; *P* < 0.001). Mild hallux valgus showed no statistically significant association with BMI (OR: 0.93, 95% CI: 0.82–1.05; *P* = 0.244) (Table 1; Figure 3). Continuous BMI-response curves showed a positive gradient for flatfoot and an inverse gradient for hindfoot valgus (Figure 4). Phenotype-combination plots showed a shift from isolated hindfoot valgus toward flatfoot-only patterns across BMI quartiles, while combined flatfoot-hindfoot patterns were comparatively stable (Figure 5). With the stricter source narrative thresholds, the hallux valgus prevalence rate was 7.3% and its BMI association remained non-significant (OR: 0.90, 95% CI: 0.77–1.05; *P* = 0.182), while the hindfoot valgus prevalence rate was 40.5% and its inverse BMI association persisted (OR: 0.81, 95% CI: 0.75–0.88; *P* < 0.001). In the one-record-per-identifier analysis (*n* = 3,266), BMI ORs were 1.38 for flatfoot, 0.94 for mild hallux valgus, and 0.82 for hindfoot valgus, materially unchanged from the primary analysis.

## Discussion

The principal finding was that BMI did not relate uniformly to the three scanner-derived foot phenotypes measured within the same school-screening workflow. A higher BMI was associated with greater odds of flatfoot, lower odds of hindfoot valgus, and no statistically significant difference in mild hallux valgus. The concurrent analysis of arch index, hallux angle, and hindfoot angle in 3,292 records is a key contribution because most pediatric BMI studies have focused on arch morphology alone. The persistence of the directions in threshold and duplicate-identifier sensitivity analyses supports the robustness of the statistical pattern, while the cross-sectional design prevents causal interpretation.

The positive association between BMI and flatfoot is consistent with systematic reviews and school- or footprint-based studies linking higher body mass with lower arches, altered plantar loading, and greater flatfoot prevalence. Potential explanations include greater mechanical loading and greater plantar soft-tissue thickness, although a footprint-derived arch index cannot distinguish structural arch lowering from soft-tissue contact. The age and sex associations in Table 1 also reinforce that pediatric foot morphology is developmentally patterned and that unadjusted BMI comparisons may be confounded.

The inverse association between BMI and hindfoot valgus was counterintuitive and must not be interpreted as a protective effect. Arch index and hindfoot angle capture different constructs: plantar midfoot contact and calcaneal frontal-plane alignment need not change in parallel. In addition, body size may influence scanner landmark detection, soft-tissue contours, or the probability of crossing an operational angle threshold without representing true anatomic realignment. Residual confounding by maturation, footwear, and lower-limb alignment may also contribute. Because the manufacturer-reported rater-reliability assessments could not be independently appraised and radiographic criterion validity has not been publicly documented for this device, the inverse association requires replication with standardized clinical and radiographic measures.

No statistically significant BMI association was observed for mild hallux valgus in either the primary or the stricter-threshold analysis. Pediatric hallux valgus has distinct developmental, anatomic, and familial determinants, and management before skeletal maturity is generally conservative, unless symptoms, progression, or underlying disorders justify referral. The present findings therefore do not support using BMI alone to identify hallux valgus risk in school screening.

The results have two practical implications. First, arch, hallux, and hindfoot measures should be reported separately rather than collapsed into a single “abnormal foot” indicator, because their associations with BMI differed in direction and magnitude. Second, a positive scanner result should prompt symptom-based clinical assessment rather than automatic diagnosis or treatment. This is particularly important for flatfoot, which was present in nearly two-thirds of records and is frequently flexible and asymptomatic in children; evidence does not support routine treatment of asymptomatic flexible flatfoot (23–26).

The strengths of the study include a large project-based school-screening dataset, concurrent bilateral measurement of three foot phenotypes, standardized on-site anthropometry, multivariable adjustment for age, sex, and school, and sensitivity analyses addressing disputed threshold boundaries and repeated identifiers.

Several limitations affect interpretation and generalizability. The cross-sectional design cannot establish temporal direction or exclude reverse causality; foot morphology may affect activity and body mass, while body mass may affect foot measurements. The seven participating schools constituted the project source population, and therefore, generalization beyond these schools should be made cautiously.

Measurement and covariate limitations are also important. Because dates of birth were unavailable, exact age in months and sex-specific WHO BMI-for-age *z* scores could not be derived reliably. The sample-specific SD and quartile cut points are population dependent, are not clinical weight-status categories, and may limit direct comparison with other populations. The original screening exclusions addressed generalized joint hypermobility, ligamentous laxity, previous foot or lower-limb injury, other deformities, and markedly atypical physical activity levels, although residual variation among eligible children was not modeled. Height and weight were measured on site with a specified instrument under standardized procedures; the analytic archive did not include calibration logs or repeat-measurement records, and therefore, any associated measurement error could not be quantified. The manufacturer reported inter- and intrarater reliability assessments, but the non-public methods and numerical results could not be independently appraised, and no publicly available radiographic criterion-validity data for the device model were identified. The operational cutoffs are method-dependent rather than universal pediatric diagnostic thresholds. Finally, 26 repeated school-student identifier pairs were present; the files could not distinguish repeat scans from identifier reuse, although the one-record-per-identifier sensitivity analysis did not materially change the BMI estimates.

Accordingly, these findings should be interpreted as associations among project-based screening measures rather than evidence that BMI causes or prevents a foot condition. Replication in prospectively sampled cohorts with documented anthropometric quality control, exact age in months, validated clinical or radiographic outcomes, independently reported device-specific reliability, relevant confounders, and longitudinal follow-up is required.

## Data Availability

The datasets presented in this article are not readily available because the data contain school- and student-level identifiers that are subject to institutional and school administrative permissions. De-identified data may be made available by the corresponding author upon reasonable request and subject to approval by Tangshan Second Hospital and the participating schools. The private school-code key will not be shared as public data.

## References

[1] Cole TJ Bellizzi MC Flegal KM Dietz WH. Establishing a standard definition for child overweight and obesity worldwide: international survey. Br Med J. (2000) 320:1240. 10.1136/bmj.320.7244.1240 PMC27365 10797032

[2] De Onis M Onyango AW Borghi E Siyam A Nishida C Siekmann J. Development of a WHO growth reference for school-aged children and adolescents. Bull W H O. (2007) 85:660–7. 10.2471/blt.07.043497 18026621 PMC2636412

[3] Carr JB Yang S Lather LA. Pediatric pes planus: a state-of-the-art review. Pediatrics. (2016) 137:e20151230. 10.1542/peds.2015-1230 26908688

[4] Banwell HA Paris ME Mackintosh S Williams CM. Paediatric flexible flat foot: how are we measuring it and are we getting it right? A systematic review. J Foot Ankle Res. (2018) 11:21. 10.1186/s13047-018-0264-3 29854006 PMC5975578

[5] Cavanagh PR Rodgers MM. The arch index: a useful measure from footprints. J Biomech. (1987) 20:547–51. 10.1016/0021-9290(87)90255-7 3611129

[6] Stolzman S Irby MB Callahan AB Skelton JA. Pes planus and paediatric obesity: a systematic review of the literature. Clin Obes. (2015) 5:52–9. 10.1111/cob.12091 25808780 PMC4631254

[7] Mickle KJ Steele JR Munro BJ. The feet of overweight and obese young children: are they flat or fat? Obesity. (2006) 14:1949–53. 10.1038/oby.2006.227 17135610

[8] Xu L Gu H Zhang Y Sun T Yu J. Risk factors of flatfoot in children: a systematic review and meta-analysis. Int J Environ Res Public Health. (2022) 19:8247. 10.3390/ijerph19148247 35886097 PMC9319536

[9] Shen J Liu J Liang F Liu X Zhang M. Prevalence of flatfoot and analysis of plantar pressure distribution in adolescents based on body mass index: a regional study. J Orthop Surg Res. (2024) 19:864. 10.1186/s13018-024-05365-9 39710719 PMC11664854

[10] Gilmour JC Burns Y. The measurement of the medial longitudinal arch in children. Foot Ankle Int. (2001) 22:493–8. 10.1177/107110070102200607 11475457

[11] Scholz T Zech A Wegscheider K Lezius S Braumann K-M Sehner S. Reliability and correlation of static and dynamic foot arch measurement in a healthy pediatric population. J Am Podiatr Med Assoc. (2017) 107:419–27. 10.7547/16-133 28708428

[12] El O Akcali O Kosay C Kaner B Arslan Y Sagol E. Flexible flatfoot and related factors in primary school children: a report of a screening study. Rheumatol Int. (2006) 26:1050–3. 10.1007/s00296-006-0128-1 16670858

[13] Chang C-H Chen Y-C Yang W-T Ho P-C Hwang A-W Chen C-H. Flatfoot diagnosis by a unique bimodal distribution of footprint index in children. PLoS One. (2014) 9:e115808. 10.1371/journal.pone.0115808 25551228 PMC4281062

[14] Abich Y Mihiret T Akalu TY Gashaw M Janakiraman B. Flatfoot and associated factors among Ethiopian school children aged 11 to 15 years: a school-based study. PLoS One. (2020) 15:e0238001. 10.1371/journal.pone.0238001 32841276 PMC7447044

[15] Lokesh M Menon PG Ashok TR. Prevalence of flat foot in south Indian school going children and concordance between Stahelis Plantar Arch Index, Chippaux Smirak Index and denis foot print grade in diagnosing flat foot. Indian J Orthop. (2025) 59:1959–68. 10.1007/s43465-025-01486-3 41245280 PMC12615885

[16] Rosende-Bautista C Munuera-Martínez PV Seoane-Pillado T Reina-Bueno M Alonso-Tajes F Pérez-García S. Relationship of body mass index and footprint morphology to the actual height of the medial longitudinal arch of the foot. Int J Environ Res Public Health. (2021) 18:9815. 10.3390/ijerph18189815 34574735 PMC8465021

[17] Seidenstein AH Torrez TW Andrews NA Patch DA Conklin MJ Shah A. Pediatric hallux valgus: an overview of history, examination, conservative, and surgical management. Paediatr Child Health. (2022) 27:75–81. 10.1093/pch/pxab074 35599675 PMC9113854

[18] Chell J Dhar S. Pediatric hallux valgus. Foot Ankle Clin. (2014) 19:235–43. 10.1016/j.fcl.2014.02.007 24878412

[19] Mahan ST Cidambi EO. Juvenile hallux valgus. Foot Ankle Clin. (2021) 26:807–28. 10.1016/j.fcl.2021.07.008 34752239

[20] Knörr J Soldado F Violas P Sánchez M Doménech P De Gauzy JS. Treatment of hallux valgus in children and adolescents. Orthop Traumatol Surg Res. (2022) 108:103168. 10.1016/j.otsr.2021.103168 34871795

[21] Kanatli U Gözil R Besli K Yetkin H Bölükbasi S. The relationship between the hindfoot angle and the medial longitudinal arch of the foot. Foot Ankle Int. (2006) 27:623–7. 10.1177/107110070602700810 16919216

[22] World Health Organization. *Physical status: the use and interpretation of anthropometry: report of a WHO Expert Committee. WHO Technical Report Series No. 854*. Geneva: World Health Organization (1995).

[23] Harris EJ Vanore JV Thomas JL Kravitz SR Mendelson SA Mendicino RW. Diagnosis and treatment of pediatric flatfoot. J Foot Ankle Surg. (2004) 43:341–73. 10.1053/j.jfas.2004.09.013 15605048

[24] Dare DM Dodwell ER. Pediatric flatfoot: cause, epidemiology, assessment, and treatment. Curr Opin Pediatr. (2014) 26:93–100. 10.1097/MOP.0000000000000039 24346183

[25] Ford SE Scannell BP. Pediatric flatfoot: pearls and pitfalls. Foot Ankle Clin. (2017) 22:643–56. 10.1016/j.fcl.2017.04.008 28779814

[26] Evans AM Rome K Carroll M Hawke F. Foot orthoses for treating paediatric flat feet. Cochrane Database Syst Rev. (2022) 2022:CD006311. 10.1002/14651858.CD006311.pub4 PMC8759438 35029841

